# Supplementary material for: Quantifying Intracellular Viral Pathogen: Specimen Preparation, Visualization and Quantification of Multiple Immunofluorescent Signals in Fixed Human Airway Epithelium Cultured at Air-Liquid Interface
Source: J Pers Med. 2022 Oct 7;12(10):1668. doi: 10.3390/jpm12101668 (PMC9605096; doi:10.3390/jpm12101668)
Supplement: Supplementary file 1 [file jpm-12-01668-s001.zip › Figure S1_Wong Pandzic_Submission.pdf]

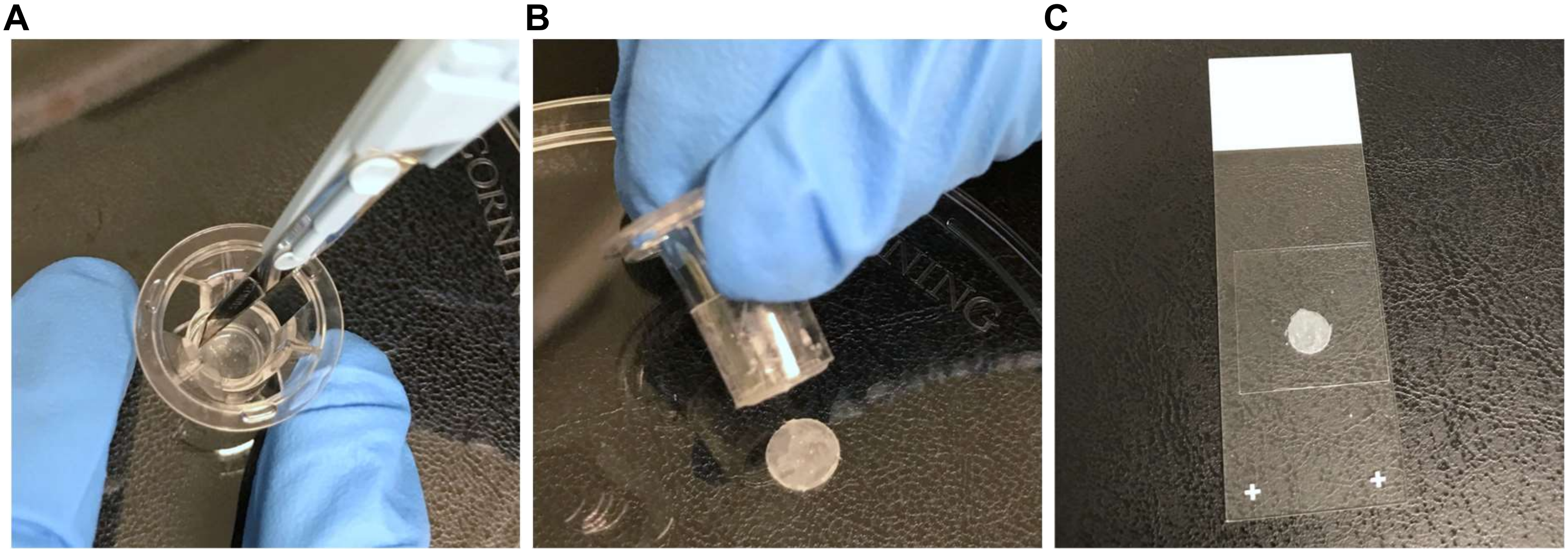

Figure S1. Excision of the air-liquid interface insert membrane prior to mounting for imaging. (A-B) Membrane is cut out from its insert using a scalpel and (C) transferred to a microscope slide with the apical side of the ALI cultures upward facing.
